# Societal pessimism and trajectories of fertility expectations among Dutch non-parents

**DOI:** 10.1186/s41118-025-00246-3

**Published:** 2025-05-12

**Authors:** Katya Ivanova

**Affiliations:** Tilburg University, Tilburg, The Netherlands

**Keywords:** Fertility expectations, Fertility intentions, Parenthood, Societal pessimism, Uncertainty

## Abstract

This study utilizes prospective data from the Dutch Longitudinal Internet Studies for the Social Sciences (LISS) panel to explore two research questions. First, can distinct trajectories of fertility expectations be identified among Dutch women and men in reproductive age (18 to 45/50) who do not have children? Second, do these trajectories differ in terms of self-reported societal pessimism at the start of that trajectory? We employed joint latent class analysis with Latent GOLD which allowed us to model the trajectories of fertility expectations while accounting for the fact that some of the respondents made the transition to parenthood during observation. The trajectories were estimated from entry into the panel until dropout, the end of the observation period (2022, or until reaching age 45 for women and 50 for men), or until the participant became a parent. For both women (*n* = 1,260) and men (*n* = 1,110), three similar classes emerged: 'committed to parenthood' (the largest class), 'uncertain' (24% of women and 22% of men), and 'early commitment to no expectation' (13.3% for women and 9.4% for men). In addition, a fourth class—'later arrivals to expectation of parenthood'—was identified among men. Bias-adjusted comparisons revealed significant differences only among women: those committed to parenthood exhibited the lowest levels of societal pessimism, while the 'uncertain' group reported the highest. These differences persisted even after adjusting for relevant control variables at the time when societal pessimism was captured, including depression levels, income satisfaction, partnership status, and education.

**Supplementary Information:**

The online version contains supplementary material available at 10.1186/s41118-025-00246-3.

## Introduction

“Why bother having a kid when the world's going to hell anyway?”

(My Year of Rest and Relaxation by Ottessa Moshfegh).

The growing concern over declining fertility rates extends beyond academic circles. Headlines such as “Suddenly there aren’t enough babies. The whole world is alarmed” (Ip & Adamy, 2024) and “The great baby bust” (The Week UK, 2024) have become commonplace in news outlets, highlighting widespread global alarm. Substantial academic research aims to understand why fewer children are being born. One line of inquiry examines the impact of structural factors, such as persistent gender inequality in the division of paid and unpaid labor (e.g., Goldscheider et al., 2015), the increasing precariousness of employment conditions (e.g., Comolli & Vignoli, 2021; Mills & Blossfeld, 2013), and housing unaffordability (e.g., van Wijk, 2024). Another research avenue, largely influenced by the Second Demographic Transition framework (Lesthaeghe, 2010; Lesthaeghe & Kaa, 1986), emphasizes cultural and ideological shifts, such as increased secularization and individualization, as crucial for understanding the potential stabilization at low fertility rates across different national contexts (see Zaidi & Morgan, 2017 for a review). This latter perspective has gained renewed attention as fertility rates continue to fall, even in gender-egalitarian countries with strong welfare states and favorable work–life policies (for example, Finland; Nordic Statistics, 2023). Against this backdrop of heightened urgency to understand what drives fertility rates, this paper explores how individuals' pessimism about the potential future of the next generation—termed here *societal pessimism*—might shape their fertility expectations. Importantly, rather than focusing on expectations at a single point in time, we focus on how these expectations evolve over time.

The concept of societal pessimism is not new in the social sciences. Defined broadly as “a feeling of a generalized negative certainty” (Bennett, 2001, p.181), it has often been measured with questions like “At the present time, would you say that, in general, things are going in the right direction or in the wrong direction, in [COUNTRY]?” (European Parliament, 2021) and “The way things are now, I find it hard to be hopeful about the future of the world” (European Social Survey, 2012). Traditionally explored within the fields of Political Science and Social Psychology as a predictor of voting behaviors (Steenvoorden & Harteveld, 2018; van der Bles et al., 2018), this construct has recently been examined in social demography to understand its impact on individual fertility behaviors (Ivanova & Balbo, 2024). This latter development is in line with theoretical arguments that when individuals make fertility decisions, they consider not only their own contemporaneous circumstances but also—their subjective visions on what the future may hold (Lappegård et al., 2022; Vignoli et al., 2020a, 2020b). In this study, I build on the latter stream of literature by exploring whether societal pessimism is linked to individuals' expectations about their future life courses, specifically regarding whether they will have children.

This paper makes two principal contributions. First, it enriches the ongoing debate concerning the extent to which adult childbearing expectations in Western societies are influenced not only by immediate personal circumstances but also by broader visions of humanity's future. Within that stream of literature, previous research has predominantly focused on how young adults' decisions about having children might be influenced by concerns over global warming (Golovina & Jokela, 2024; Helm et al., 2021; McMullen & Dow, 2022; Powdthavee et al., 2024; Rackin et al., 2023). However, it is critical to note that environmental concerns are often polarized along political and educational lines (Poortinga et al., 2011). This study broadens this perspective, considering a wider scope of factors affecting the future of the next generation, including the quality of social relationships and personal well-being.

Second, I align my work with literature that recognizes uncertainty in fertility expectations as a legitimate response category, one that cannot be overlooked or simply dismissed as missing data (Barker & Buber-Ennser, 2024; Kuhnt et al., 2021; Ni Bhrolchain & Beaujouan, 2015; Trinitapoli, 2023). I examine whether societal pessimism is associated with an outright expectation of remaining child-free or rather, with uncertainty about future fertility plans. This approach can offer insight into whether the previously documented link between societal pessimism and the transition to parenthood (Ivanova & Balbo, 2024) might be because of active decisions to forego parenthood, or rather because of increased hesitation about whether to become a parent.

Previous studies have explored why individuals adjust their fertility expectations over time, focusing on how changes in one’s own life—such as partnership, educational, or career transitions—relate to shifts in these expectations (e.g., Iacovou & Tavares, 2011; Liefbroer, 2009). This study introduces a novel angle by moving the focus from own circumstances to how people think about the world around them. Specifically, I aim to assess whether perceptions of the next generation's future are associated with fertility expectation trajectories. It is important to note that this study does not aim to investigate the mechanisms behind any potential association and remains explorative in nature. In other words, I do not examine whether societal pessimism influences changes in fertility expectations by affecting factors such as the likelihood of entering a stable partnership, for example. Rather, the work serves as an initial step in determining whether a link exists at all between individuals’ views of society’s future and their fertility expectations.

I use data from the Dutch Longitudinal Internet Studies for the Social Sciences (LISS) panel and address two key research questions. First: can we identify distinct, meaningful trajectories of fertility expectations among Dutch women and men in reproductive ages who do not have children? Second: can the potential discrete trajectories be linked to the respondents' level of societal pessimism at their entry into the panel? The Netherlands provides a compelling context to explore these questions, as the normative expectation to have children has significantly declined in recent decades (Noordhuizen et al., 2010), potentially fostering more diverse fertility trajectories shaped by individual preferences and future visions.

## Background

### Fertility expectations: what are they?

At the core of this study is the focus on individuals' fertility expectations. The terms “fertility expectations” and “fertility intentions” have sometimes been used interchangeably in the literature (Gemmill, 2019; Hayford, 2009; Ni Bhrolchain & Beaujouan, 2015) as previous works have asserted that the constructs are closely connected empirically (Iacovou & Tavares, 2011; Philipov & Bernardi, 2012). Fertility expectations and intentions have garnered significant attention due to their potential to predict actual behaviors (Morgan & Rackin, 2010; Ni Bhrolchain & Beaujouan, 2015; Rackin & Bachrach, 2016). At the same time, despite the close links between the two, we should acknowledge that they do not completely overlap.

One can think of intentions as much more closely related to a “commitment to act” (Rackin & Bachrach, 2016, p.531). As such, these intentions can be more strongly influenced by the concurrent structures in which people are embedded (e.g., employment conditions, partnership status, housing situation, etc.). Empirically, intentions have often been measured by also giving respondents a specific timeframe as a reference (e.g., “Do you intend to have a/another child during the next three years?” in the Generations and Gender Survey). Fertility expectations on the other hand can be seen as “a representation of a future state that is perceived to be most likely” (Rackin & Bachrach, 2016, p.531). This definition implies that expectations involve a more generalized vision of one’s life course, drawing on broader conceptions of family, self, and the future, without requiring a concrete plan. As such, fertility expectations are an interesting construct to examine even among younger respondents who may not (yet) be in the position to make specific fertility plans.

In this study, I gauge fertility expectations by asking respondents, “Do you think that you will have children in the future?” with answer options “Yes”, “No”, and “I don’t know”. Capturing these expectations at multiple timepoints complicates the prediction of developmental trajectories, as it is challenging to determine a priori which patterns will emerge. Yet, some general expectations can be stated. Given the normative expectation of parenthood as a key adult experience (Ashburn-Nardo, 2017; Brewster & Snow, 2022), it is likely that the majority of respondents will exhibit stable positive expectations over time, possibly shifting towards uncertainty or no expectations at older ages. However, the context of this study must also be considered. With increasing secularization and greater normative acceptance of childlessness in the Netherlands over recent decades (Gielens & Muis, 2022; Noordhuizen et al., 2010), I anticipate finding a significant proportion of the population deviating from traditional fertility expectation trajectories.

### Fertility expectations and societal pessimism: why would they be related?

Similar to the public’s concern with declining fertility rates noted at the outset of this paper, the rationale behind (abstaining from) parenthood, particularly in the context of the various crises facing (Western) societies, has also come under public scrutiny. Media outlets frequently feature headlines such as “Given the state of the world, is it responsible to have kids?” (O’Grady, 2019), “Should I have children? Weighting parenthood amid the climate crisis” (Bergman, 2021), and “I do not dare to bring a child into this world” (Leijssen & Ybema, 2021). When it comes to the systematic empirical study of individuals’ reasoning about their childbearing intentions, a lot of what we know stems from qualitative work among childless/childfree adults (Agrillo & Nelini, 2008).

Key factors highlighted in these studies include the anticipated effects of having children on various life aspects, worries about passing on undesirable genetic characteristics, doubts about one's ability to parent effectively, and a general lack of interest in children (Agrillo & Nelini, 2008; Park, 2005). These factors are linked by their focus on the decision-maker's perspective, either concerning the potential child's influence on the adult's life or the adult's potential impact on the child. Yet, that research also indicates that decisions about parenthood incorporate broader, community-focused considerations, such as worries about overpopulation and the overall condition of the world (Agrillo & Nelini, 2008; Park, 2005). Interestingly, some qualitative work has suggested that these reasons are more often brought forward by women than men (Park, 2005). Societal pessimism—or the general expectation that things are not going in the ‘right’ direction in society as a whole—can be seen as fitting in this latter category of reasoning. In a setting where pronatalism norms are weakened, such as the Netherlands (Noordhuizen et al., 2010), considerations about the prospective future of a potential child could have a more meaningful impact in shaping individuals’ fertility expectations.

Since I do not begin with predetermined hypotheses regarding the trajectories of fertility expectations, it is challenging to predict which trajectories will exhibit higher or lower levels of societal pessimism. However, based on the substantive arguments concerning how future concerns might influence fertility-related reasoning, I expect that the lowest levels of societal pessimism will be observed among individuals who report positive fertility expectations consistently. How societal pessimism varies among other potential fertility trajectories remains an open question.

## Methods

### Data and analytical sample

The data utilized in this study originates from the Dutch Longitudinal Internet Studies for the Social Sciences (LISS) panel, managed by CentERdata at Tilburg University, the Netherlands (https://www.dataarchive.lissdata.nl). The LISS panel uses a true probability sample from separate private households. Its sampling framework is based on the comprehensive national address directory held by Statistics Netherlands. This database, which includes addresses and municipality codes, is annually updated by Statistics Netherlands. They select a 10% random sample of the population from the municipal personal records database (Gemeentelijke Basisadministratie, GBA). The initial panel recruitment was conducted in 2007, when Statistics Netherlands, in partnership with LISS, chose 10,150 addresses from this database. The initial approach to potential participants was made through a mailed letter, providing a 10-euro incentive with no conditions attached. Following this, contacts were either made via telephone—if the number was available—or through direct home visits for in-person interviews.

In the initial meeting, participants were invited to join the LISS panel. For those who lacked the necessary technology, a computer and Internet access were provided to enable participation. Out of the entire initial sample, 48% agreed to register for the panel. At the outset in 2008, certain demographics such as single individuals, people aged over 65, university graduates, and residents from the most and least urbanized areas were less represented compared to the general population statistics reported by Statistics Netherlands (Knoef & de Vos 2009). Participants are required to fill out monthly online surveys that cover various fundamental topics, including family dynamics, personal traits, and financial conditions, with certain key surveys conducted annually. They receive financial compensation for their time. Annually, the panel experiences a dropout rate of about 12%. To maintain representativeness, new samples are periodically integrated into the panel. For more information on the LISS panel's methodology, including response rates and attrition details, see Scherpenzeel (2009, 2011), Scherpenzeel and Bethlehem (2011), and Scherpenzeel and Das (2011).

The analytical sample for this study was defined in three consecutive steps. First, respondents were selected if they filled out the "Initial Questionnaire" module of the LISS, which is administered once to new panel members when they first join. Starting in 2010, this questionnaire began to include a series of questions that probe the participants' views on the living conditions of the coming generation. These questions are used to create the societal pessimism measure explained in detail below. Between 2010 and 2021, a total of 7,911 individuals completed this survey (the last year that data on fertility transitions and expectations were available at the time of the submission of this manuscript was 2022).

Second, given the focus on how fertility expectations develop, I needed information on the “fertility expectations” question which is included in an annual module of LISS (more information below). This question is only administered to female respondents under the age of 45 and male respondents under the age of 50. Of the 7,911 participants who reported on the Initial Questionnaire, 4,985 were also within the age range to report on fertility expectations when they entered the panel and of those, 3,996 provided such a report at least once between 2010 and 2022. A comparison of the respondents who provided information on both the “Initial Questionnaire” and the fertility expectations and those who only filled out the “Initial Questionnaire” showed that the self-reported societal pessimism was significantly lower among respondents who only filled out the starting questionnaire (*M* = 3.94, *SD* = 1.04 for that group vs *M* = 4.05, *SD* = 0.91 for respondents with a measure on fertility expectations).

The third and final step in defining the analytical sample was to select respondents who were not parents at the moment when they reported on societal pessimism (i.e., their entry into LISS) and who provided at least one report on their fertility expectations during their observation window (see Analytical approach). This rendered an analytical sample of 2,359 respondents (53.4% female), who participated in an average of 3.12 data collections (*SD* = 2.27) over approximately 4.11 years (*SD* = 2.17). The average age of the respondents at first observation was 26.4 years (*SD* = 7.49). Descriptive information about the sample is provided in Table 1.

**Table 1:** Descriptive statistics for analytical sample

	Women (*n* = 1,260)	Men (*n* = 1,110)
Societal pessimism	4.08 (0.87)	3.95 (0.95)
Fertility expectation at first observation		
Positive	65.7%	58.1%
Negative	10.8%	13.7%
Unsure	23.5%	28.2%
Age at first observation (at societal pessimism)	25.42 (6.86)	27.52 (8.01)
Number of observations	3.20 (2.29)	3.04 (2.25)
Controls measured at entry into panel (i.e., timing of societal pessimism measure)		
Depression (1–6)	2.71 (0.84)	2.46 (0.84)
Satisfaction with income (0–10)	6.03 (2.04)	6.11 (2.11)
Has a partner	54.8%	51.6%
Educational level (1 = *primary school* to 6 = *university*)	3.42 (1.74)	3.51 (1.70)

### Measures

#### Societal pessimism

The principal variable of interest was defined through a series of questions included in the "Initial Questionnaire" module, which new members of the LISS panel complete only once—upon their entry into the LISS—starting from 2010. Participants were instructed that they will see a series of screens, each focusing on a different life domain (three questions per domain). For each, they were asked to evaluate how they think the living conditions for the coming generation will compare to the present. The answer scale ranged from 1 (significantly worse than today) to 7 (significantly better than today), with a neutral option at 4 (the same as today). An "I don’t know" response option was provided, which we recorded as missing data. The thematic areas covered in this contribution are: social relationships (e.g., social involvement), economic prospects (e.g., financial flexibility), social mobility and inequality (e.g., access to housing), employment (e.g., career options), and personal well-being (e.g., sense of well-being).

**Fig. 1:**
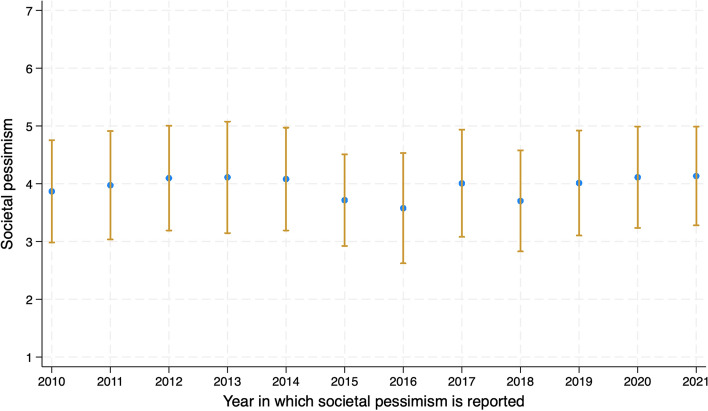
Average societal pessimism (future of coming generation is 1 = *much better* to 7 = *much worse than today*), by year of entry into panel and thus—report of societal pessimism

A composite scale was created by reverse coding and taking the average of the 15 items (so that a higher value corresponded to higher societal pessimism). The validation of the scale is discussed extensively in Ivanova and Balbo (2024). In the interest of brevity, I will not repeat the full information here, but it is important to note that the composite scale used here has high internal consistency with Cronbach’s alpha ranging between 0.87 and 0.92 across 2010 and 2021 for the respondents who were of reproductive age when they entered the LISS panel. The mean level of societal pessimism was 4.02 (*SD* = 0.91). Figure 1 gives an impression of the average level of societal pessimism across the years in which respondents joined the panel.

#### Fertility expectations

The expectations of the respondents about whether or not they will have children in the future were captured via the annually presented question, “Do you think that you will have [more] children in the future?” The answer options were “Yes”, “No” and “I don’t know”. At first observation 62.2% of the respondents declared that they expected that they will have children in the future, 12.1% did not have such an expectation, and 25.7% were unsure.

#### Transition to parenthood

Respondents reported on whether they had any (new) children at each wave of LISS. This information—alongside the annual information on expectations—was used in the joint latent class analysis as described below. During the period of observation, 260 transitions to parenthood were captured.

#### Time-invariant control variables

When inspecting the association between societal pessimism and the latent classes, several potential confounders in this association were accounted for. Those confounders were captured at the entry into the panel (i.e., the moment when societal pessimism was measured as well). Further information as to why I do not consider time-varying control variables is provided in the analytical approach section.

I accounted for self-reported depressive feelings which were operationalized by presenting the respondents with 5 items (e.g., I felt depressed and gloomy) and asked to indicate the answer that best described how they felt in the past month on a scale from 1 = *never* to 6 = *continuously*. I also included controls for satisfaction with own financial situation (i.e., “How satisfied are you with your financial situation?”, 0 = *not satisfied at all* to 10 = *entirely satisfied*), current educational level (captured using the categories of Statistics Netherlands ranging from 1 = *primary school* to 6 = *university*), and whether or not the respondent had a partner at entry into the panel. Table 1 provides the descriptive statistics for these variables.

### Analytical approach

I used Latent GOLD 6.0 software (Vermunt & Magidson, 2021) to carry out a joint latent class analysis, where I simultaneously modeled the discrete-time hazard rate of having the first child alongside fertility expectations. The latent classes were differentiated by both the fertility expectation trajectories and the likelihood of having a first child at specific ages, assuming no prior childbirth. I employed binary logistic regression for the variable “transition to parenthood” using age as the predictor. The transition to parenthood—though not the focus in this contribution—is included in the estimation of the trajectories as respondents were censored after having their first child (further details on censoring are provided below). In other words, missing values on fertility expectations after this transition are not at random. This method, often termed joint modeling, is typically used to analyze a longitudinal outcome together with a time-to-event variable (Proust-Lima et al., 2014). For further details on how Latent GOLD manages missing data, refer to Vermunt and Magidson (2016).

**Fig. 2:**
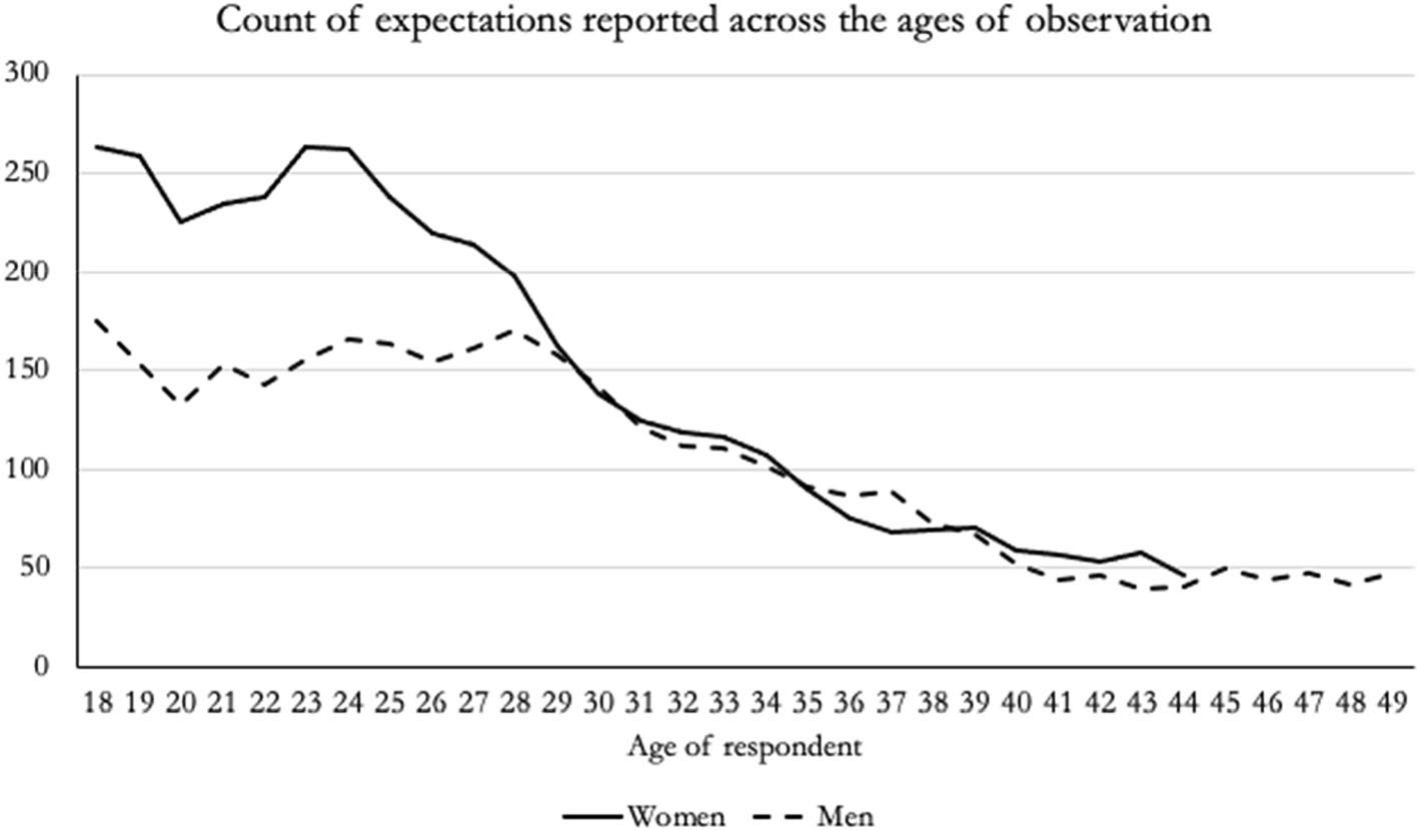
Count of fertility expectations across the observation age window

The joint latent class model included regression analyses for fertility expectations and the first-birth hazard rate, using age as the key predictor. I applied nominal logistic regression to model fertility expectations and binary logistic regression for the first-birth hazard rate. Individuals were observed from entry into the panel until (1) end of observation period (2022); (2) they dropped out of LISS; (3) they passed the age of 45 for women and 50 for men; or (4) they became parents. The distribution of how many observations of fertility expectations were available across the age range is provided in Fig. 2. Age dependence was modeled using a b-spline approach, which employs piecewise polynomials that connect at predetermined knots—these knots were evenly distributed across the observed age range (Vermunt & Magidson, 2021). Specifically, I incorporated cubic age effects with an intermediate knot, resulting in a model configuration that included four distinct age parameters. In this model, I allowed the regression coefficients, including intercepts and age effects, to vary across different latent classes, enabling a nuanced analysis of how these trajectories differ among subgroups within the population.

I conducted a series of estimations, progressively increasing the number of classes while ensuring a sufficiently large number of initial values to circumvent local maxima. The optimal number of classes was identified based on the lowest Bayesian Information Criterion (BIC), which provides a heavier penalty for the number of parameters than the Akaike Information Criterion (AIC; Schoot et al., 2017). In addition, I analyzed the bivariate residuals for the longitudinal data to assess the effectiveness of the model in capturing the overall associations and the autocorrelations between consecutive timepoints. Finally, the theoretical coherence of the identified classes was taken into account, ensuring distinctiveness between trajectories and maintaining a minimum class size of at least 5% of the sample to validate each trajectory as a separate class. This comprehensive approach ensured a robust model that is both statistically sound and theoretically substantiated. Once the optimal number of classes was determined, I applied a bias adjusted three-step approach to examine whether these classes differed significantly from each other with respect to societal pessimism. The advantage of this approach is that it takes misclassification into account (see Vermunt, 2010). I estimated trajectories separately for men and women, acknowledging the gendered impact of parenthood on life trajectories (Nomaguchi & Milkie, 2020) and its potential influence on (changes in) parenthood expectations over the life course. In addition, the enduring normative expectation that motherhood is central to women's identities—often referred to as 'the motherhood mandate' (Russo, 1976)—further justifies the split analyses.

As the reader will note, the analyses do not include any time-varying covariates (besides current age of the respondent in the class specification stage). Of course, it is possible that how expectations adjust over time can be explained by numerous contemporaneous individual circumstances (e.g., current employment and partnership status). However, the goal of this contribution is not to explain why people adjust their expectations upwards or downwards. The twofold focus of this contribution is on (1) providing a description of the predominant patterns of fertility expectations in the population of interest; and (2) examining if the identified groups differ in their level of societal pessimism as reported at the entry into the LISS panel. Therefore, given that the key predictor is measured only once—at the entry into the LISS panel—I do not consider time-varying controls such as change in partnership or employment status. The inclusion of the controls was guided by the ambition to correct for possible confounding factors which could be influencing both how individuals perceive the future of the coming generation, as well as their own fertility expectations.

## Results

### Classes on fertility expectations: men

**Fig. 3:**
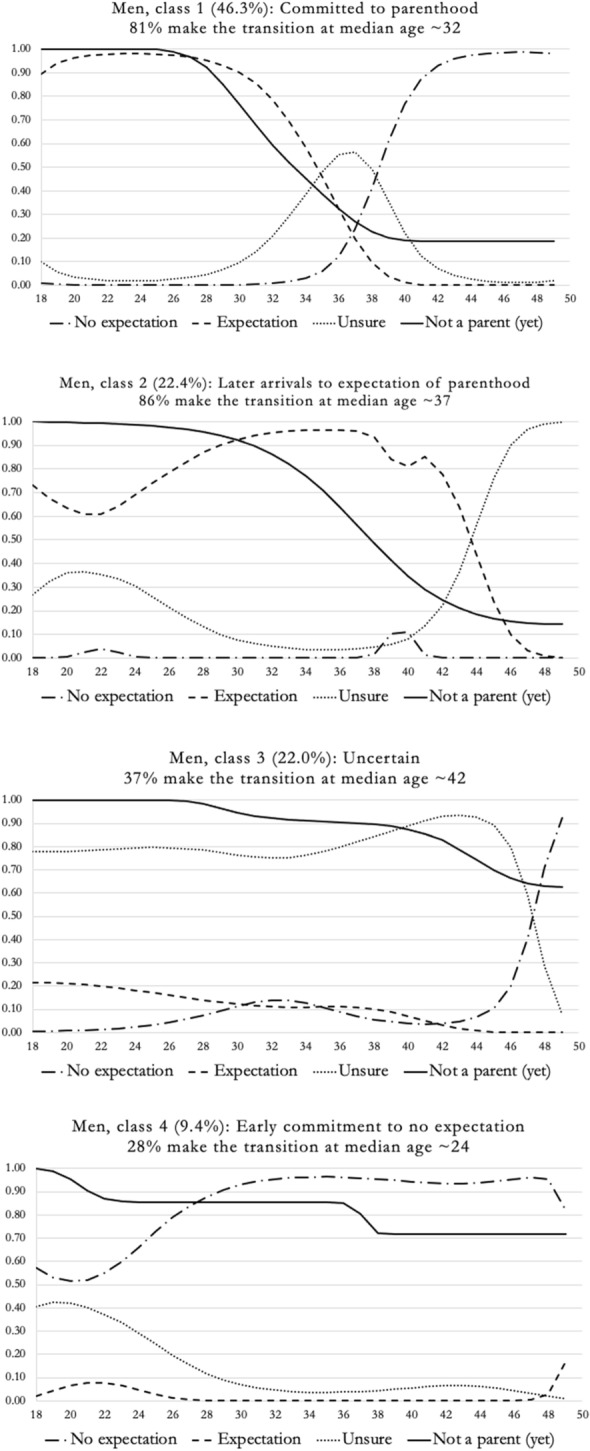
Fertility expectation trajectories of men (4 class solution)

For men, the joint latent class analysis showed that the four-class model performed best, based on the lowest BIC value, decreased autocorrelation in fertility expectations, large enough and interpretable classes. Table 2 displays the fit statistics for models with one-to-five classes. Table A1 in the supplemental materials displays the regression coefficients for the four-class model. However, since these coefficients are challenging to interpret, the classes are visually represented in Fig. 3.

**Table 2:** Model fit statistics for the 1–5 class models for men

	BIC(LL)	AIC(LL)	AIC3(LL)	BVR (Lag1–Lag2)	VLMR	BLRT	Entropy R^2^	Class proportion
1-class	6735.78	6660.74	6675.74	107.87–109.59			1.00	
2-class	5688.98	5533.88	5564.88	21.00–22.96	1158.85 (*p* < .05)	*p* < .001	0.71	.64/.36
3-class	5495.01	5259.87	5306.87	4.66–5.85	306.02 (*p* < .05)	*p* < .001	0.66	.58/.30/.12
**4-class**	**5484.92**	**5169.73**	**5232.73**	**1.61–2.67**	**122.14** **(*p* < .05)**	***p* < .001**	**0.55**	**.46/.22/.22/.09**
5-class	5522.23	5125.99	5204.99	1.59–3.08	75.75 (*p* < .05)	*p* < .001	0.52	.41/.22/.18/.10/.09

*AIC* Akaike information criterion, *BIC* Bayesian information criterion, *VLMR* Vuong–Lo-Mendel–Rubin test, *BLRT* bootstrap likelihood-ratio test, *BVR (Lag1–Lag2)* Longitudinal bivariate residuals for fertility expectations

Number of starting values: sets = 750, iterations = 1500 for men. In bold—chosen class

The largest class among men (46.3%) can be labeled as "*committed to parenthood*." This trajectory is characterized by early expectation of having children. This expectation shifts to uncertainty between the ages of 30 and 40, followed by a rapid increase in having no such expectation from age 36 onwards. A significant majority of these men (81%) became fathers, around the median age of 32.

The second largest group (22.4%) was characterized by a high proportion of men transitioning to parenthood (86% of the class). Initially, this group exhibited higher levels of uncertainty until about the mid-20 s, which then shifted to a clearly increased expectation of having children, maintained until the early 40 s. The median age for becoming fathers in this group was approximately 37, thus it was labeled "*later arrivals to expectation of parenthood*."

The third class, similar in size to the second (22%), showed a distinct pattern of expectation development compared to classes 1 and 2. This group maintained stable uncertainty throughout the observation period regarding future parenthood. By the end of the period, this uncertainty shifted to no expectation. A considerable proportion (37%) still transitioned to parenthood, though at a later median age of approximately 42. This group was labeled "*uncertain*."

The final class, comprising 9.4% of the men, was characterized by an early and steep rise in having no expectations of having children. Only a minority of these men (28%) made the transition to parenthood, doing so at a relatively young median age of about 24. This group was labeled "*early commitment to no expectation*."

### Classes on fertility expectations: women

**Fig. 4:**
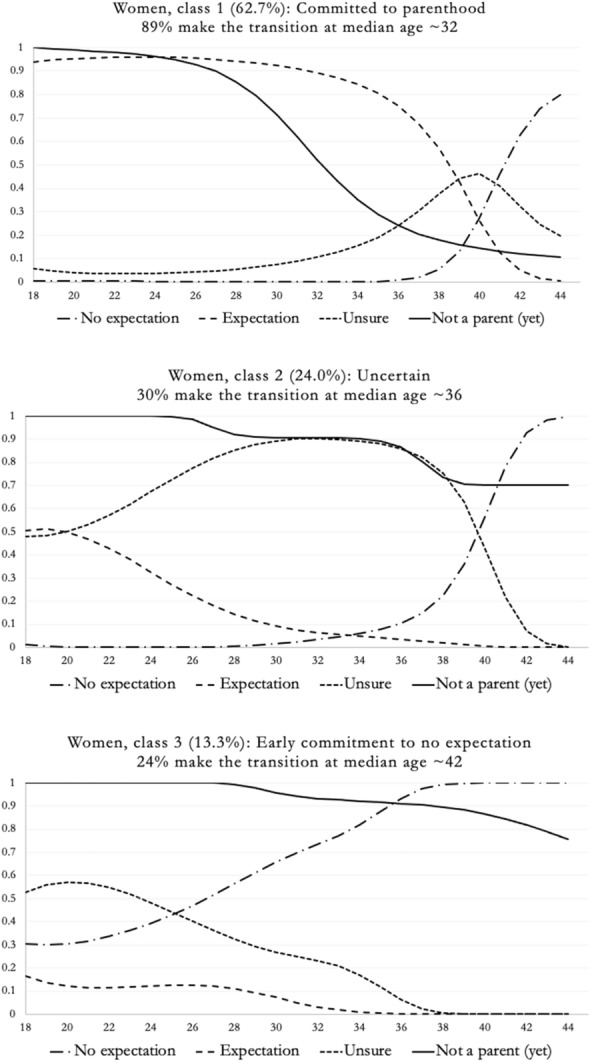
Fertility expectation trajectories of women (3 class solution)

For women, the joint latent class analysis showed that the three-class model performed best, based on the lowest BIC value, decreased autocorrelation in fertility expectations, large enough and interpretable classes. Table 3 displays the fit statistics for models with one-to-five classes. Table A2 in the supplemental materials displays the regression coefficients for the three-class model and the classes are plotted in Fig. 4.

**Table 3:** Model fit statistics for the 1–4 class models for women

	BIC(LL)	AIC(LL)	AIC3(LL)	BVR (Lag1–Lag2)	VLMR	BLRT	Entropy R^2^	Class proportion
1-class	7350.253	7273.17	7288.17	98.60–111.37			1.00	
2-class	6151.672	5992.368	6023.368	16.87–21.98	1312.80 (*p* < .05)	*p* < .001	0.70	.66/.34
**3-class**	**6006.017**	**5764.491**	**5811.491**	**7.03–10.44**	**259.88** **(*p* < .05)**	***p* < .001**	**0.65**	**.63/.24/.13**
4-class	6029.987	5706.238	5769.239	4.57–7.23	90.25 (*p* < .05)	*p* < .001	0.62	.61/.19/.15/.05

*AIC* Akaike information criterion, *BIC* Bayesian information criterion, *VLMR* Vuong–Lo-Mendel–Rubin test, *BLRT* bootstrap likelihood-ratio test, *BVR (Lag1–Lag2)* Longitudinal bivariate residuals for fertility expectations

Number of starting values: sets = 1000, iterations = 1750 for women. In bold—chosen class

Similar to the men, the largest class among women (62.7%) is labeled "*committed to parenthood*", where expectation of having children in the future is displayed from the start of the trajectory. This expectation shifted to uncertainty in the late 30 s, followed by a rapid increase in having 'no expectation' from age 40 onwards. A significant majority of these women (89%) became mothers, typically around the median age of 32.

The second largest group (24%) resembled the "*uncertain*" class observed among men. This group displayed high uncertainty throughout the observation period whether they would have children. From the late 30 s, this uncertainty transitioned to no expectation. Notably, a considerable proportion (30%) of this group still made the transition to parenthood, albeit at a somewhat later median age of approximately 36.

The final class, comprising 13.3% of the women in the study, was characterized by initial uncertainty followed by a steady increase in having 'no expectations' of having a child. About a quarter of the women in this class (24%) transitioned to parenthood, though at a much later median age than the other clusters (approximately 42). This group was labeled "*early commitment to no expectation*," consistent with the men’s group.

In summary, three similar classes could be identified for both the men and the women in our sample: “*committed to parenthood*” (the largest class for both), “*uncertain*” (about a fifth of the sample for both), and “*early commitment to no expectation*” (9.4–13.3% of the samples). For men, an additional class of about a fifth of the sample could be identified and was labelled “*later arrivals to expectation of parenthood*”.

### Differences between classes on societal pessimism at entry into panel

As specified in the analytical approach, once the optimal number of classes was determined, I could apply the bias adjusted three-step approach to examine if the identified classes differed on the self-reported societal pessimism at entry into the panel. The differences were examined in two steps: first by only accounting for the respondents’ ages at the report of societal pessimism and then while also controlling for level of depression, satisfaction with income, educational level, and partnership status at entry into the panel.

For men, the analyses which included only a control for age at entry into panel demonstrated that the classes differed significantly from each other on societal pessimism (Wald = 11.12, *p* < 0.05). Pairwise comparisons showed that the significant differences in societal pessimism were between classes one (*M*_societal pessimism_ = 3.90) and four (*M*_societal pessimism_ = 4.25; Wald = 5.39, *p* < 0.05), classes two (*M*_societal pessimism_ = 3.82) and three (*M*_societal pessimism_ = 4.01; Wald = 4.28, *p* < 0.05), and classes two and four (Wald = 7.18, *p* < 0.05). In summary, class four—the early committers to ‘no expectation’—exhibited the highest levels of societal pessimism. They were closely followed by class three—the ‘uncertain’ group. Class two—the later arrivals to expectation of parenthood—scored the lowest on this indicator, closely followed by class one—the early commitment to parenthood class (*M*_societal pessimism_ = 3.89). However, these differences were no longer significant once I controlled for the respondents’ characteristics at entry into the panel.

For women, both the analyses which included only a control for age at entry into panel, as well as the ones with the full set of controls demonstrated that the classes differed significantly from each other on societal pessimism (Wald = 7.79, *p* < 0.05). In the interest of succinctness, I will only discuss the results with the full controls.^1^ Pairwise comparisons showed that the significant difference in societal pessimism was only between class one—the committed to parenthood (*M*_societal pessimism_ = 3.96) and class two—the uncertain group (*M*_societal pessimism_ = 4.21; Wald = 4.73, *p* < 0.05). The early commitment to no expectation group scored somewhat lower than the uncertain group (*M*_societal pessimism_ = 4.18). The finding that societal pessimism is lowest for the trajectory with stable expectation of parenthood is in line with the tentative expectation stated at the end of the theoretical section. Table 4 displays the mean levels of the different characteristics across the classes, as well as the significant contrasts.

**Table 4:** Differences in societal pessimism (and key control variables) between class of women

	1 vs 2	1 vs 3	2 vs 3
	Wald value	Wald value	Wald value
Societal pessimism (*M*_1_ = 3.96, *M*_2_ = 4.21, *M*_3_ = 4.18)	**4.73** **(*p* < .05)**	2.09 (*p* > .05)	0.09 (*p* > .05)
Age at first observation (*M*_1_ = 24.16, *M*_2_ = 25.43, *M*_3_ = 26.30)	**10.80** **(*p* < .01)**	**25.29** **(*p* < .01)**	2.40 (*p* > .05)
Depressive feelings at first observation (*M*_1_ = 2.64, *M*_2_ = 2.86, *M*_3_ = 2.91)	2.89 (*p* < .10)	**4.52** **(*p* < .05)**	0.28 (*p* > .05)
Satisfaction with income at first observation (*M*_1_ = 6.19, *M*_2_ = 5.67, *M*_3_ = 6.00)	3.05 (*p* < .10)	0.00 (*p* > .05)	1.56 (*p* > .05)
Has a partner at first observation (*M*_1_ = 0.63, *M*_2_ = 0.36, *M*_3_ = 0.43)	**24.33** **(*p* < .01)**	**12.35** **(*p* < .01)**	1.30 (*p* > .05)
Educational level at first observation (*M*_1_ = 3.59, *M*_2_ = 3.30, *M*_3_ = 3.09)	3.79 (*p* = .05)	**15.64** **(*p* < .01)**	3.18 (*p* < .10)

*M*_1_ = mean for “committed to parenthood” class, *M*_2_ = mean for “uncertain” class, and *M*_3_ = early commitment to no expectation” class. “First observation” is the moment when societal pessimism is measured. Significant differences are presented in bold

## Discussion

This work set out to answer two key research questions. First, can distinct trajectories of fertility expectations be identified among women and men in reproductive ages who do not have children? Second, do these potential discrete trajectories differ with respect to the individuals’ level of pessimism about the future of the next generation? Based on the analyses of Dutch panel data, three main conclusions can be drawn from this work.

Primarily, the expectation trajectories which were identified were similar for men and women. As anticipated, the largest group was a developmental trajectory that appears quite normative. Within this group, individuals had positive expectations about having children from early on and throughout the reproductive years. These expectations shifted to 'uncertain' at the start of the 30s and then to 'no expectation' by the late 30s. Notably, the vast majority of individuals in this cluster transitioned to parenthood. Given the primacy of parenthood in the adulthood experience (Ashburn-Nardo, 2017; Brewster & Snow, 2022), the dominance of this group is not surprising. Interestingly, about one in five individuals from both genders consistently exhibited uncertainty about having children, starting at an early age. This suggests that uncertainty is not merely a transitional phase between positive and negative expectations but can also represent a stable state of expectation. This pattern supports research into pathways into childlessness, which describes “prolonged ambivalence” (Gemmill, 2019, p.132) as a significant factor. Most individuals in this uncertain category did not become parents during the study period. An even lower rate of parenthood was observed among those who, from a relatively early age onwards, declared no expectation of parenthood (about 10% of men and 13% of women). What set this group apart from the prolonged ambivalence described above is the early emergence of ‘no expectation’ with respect to having children. While we cannot speak to the motivations behind this expectation, this early commitment to ‘no expectation’ can potentially be seen as indicative of a general disinterest in children or of a preference for a lifestyle that is incompatible with having children and potentially especially so for women (Hakim, 2003). It is worth noting that the commitment to ‘no expectation’ in this trajectory was more pronounced at an early age among men compared to women, who initially exhibited greater uncertainty and shifted to ‘no expectation’ in their mid-20s. This difference is unsurprising, given the normatively endorsed centrality of motherhood in women’s adult lives (Russo, 1976), which may make it more challenging for women to articulate childfree intentions early on.

The second key observation relates to the association between fertility expectation trajectories and self-reported societal pessimism upon entering the panel. The findings suggest a link between individuals’ fertility expectations and their perceptions of the future facing potential children. Those in the ‘committed to parenthood’ trajectory, who maintained consistently positive fertility expectations, reported the lowest levels of societal pessimism at their initial assessment, with those in the ‘uncertain’ and the ‘early commitment to no expectation’ scoring higher on societal pessimism. This recognition of the important role which the “shadow of the future” (Bernardi et al., 2019, p.4) can play in how individuals think about and make family-related decision in the present is certainly not new. This study contributes to the expanding body of research demonstrating that perceptions of personal (financial) uncertainty (e.g., Comolli, 2017; Comolli & Vignoli, 2021; Trinitapoli, 2023) and broader societal concerns (e.g., Golovina & Jokela, 2024; Helm et al., 2021; Ivanova & Balbo, 2024; McMullen & Dow, 2022; Powdthavee et al., 2024; Rackin et al., 2023) can adversely affect fertility intentions and behaviors. Notably, the correlation between societal pessimism and fertility trajectories was robust only among women, aligning with previous findings that childless women more frequently cite collective-oriented motivations (Park, 2005). Exploring the roots of these gender differences remains outside the scope of this paper but represents a promising direction for future research into decision-making processes under conditions of uncertainty or negative certainty among men and women.

The final observation concerns the pathways that could lead women reporting higher levels of societal pessimism to forego fertility. Interestingly, the highest levels of societal pessimism were not found among those who early on committed to “no expectation” of having children, but rather within the “uncertain” group. It is important to approach this finding with caution, as the average level of societal pessimism between these two groups was rather similar. Yet, this distinction is intriguing, especially considering research indicating that young people who report an intention to remain childfree often cite “just don’t want children” as their primary motivation (Brown, 2021). Thus, while societal pessimism might not directly drive the decision to remain childfree, its association with “prolonged ambivalence” about having children (Gemmill, 2019, p.132) may still lead to ultimately foregoing parenthood.

Of course, the findings presented must be viewed within the context of the study's limitations. Primarily, the research is descriptive, providing insights into the fertility expectation trajectories among Dutch men and women and their association with initial levels of societal pessimism. However, it does not explore the specific mechanisms behind these trajectories, such as how changes in personal circumstances like relationship dynamics—which may themselves be influenced by societal pessimism—impact these trajectories. Although I controlled for potential baseline confounders, I did not include time-varying covariates, as this was beyond the scope of this study. In addition, these trajectories are based on partial observations of individuals’ reproductive careers rather than their entire reproductive periods. While ideally, we would track fertility expectations throughout the full reproductive lifespan, no existing long-term prospective study on fertility expectations includes a reliable measure for societal pessimism. Moreover, analyzing complete reproductive periods would inherently focus on older cohorts, whereas the goal here was to provide a more contemporary snapshot. Finally, the trajectories identified might be highly specific to the Dutch context. In a different societal setting, particularly one with stronger pronatalist norms, we might observe very different developmental trajectories. It remains an open question whether the findings extend to similar contexts, and further replications are encouraged.

This study did not aim to compare which type of uncertainty—whether about one’s own future or the future of the next generation—has greater predictive power for fertility expectations. Instead, the focus stemmed from a growing body of empirical research suggesting that the rationale behind individual fertility decisions extends beyond a (rational) analysis of one’s immediate circumstances (e.g., see work on climate change concerns and the transition to parenthood; Powdthavee et al., 2024). Particularly in regions where having children is increasingly viewed as a personal choice rather than a normative expectation, considerations about the future that children will inherit may play an interesting role in shaping individual fertility expectations.

## Supplementary Information

Supplementary material 1.

## Data Availability

In this paper, we make use of data from the LISS panel (Longitudinal Internet studies for the Social Sciences) managed by the non-profit research institute Centerdata (Tilburg University, the Netherlands). The data archive can be accessed via https://www.lissdata.nl/use-the-data. The data files for the analyses displayed here were downloaded in November 2024.

## Footnotes

1 Supplemental analyses were run including self-rated health at the entry into LISS as an additional control (captured with “How would you describe your health, generally speaking?” 1 = *poor* to 5 = *excellent*). The results displayed here do not change and self-rated health does not differ significantly across the trajectories (Wald = 3.58 p > .05).
