# Supplementary material for: Societal pessimism and trajectories of fertility expectations among Dutch non-parents
Source: Genus. 2025 May 12;81(1):10. doi: 10.1186/s41118-025-00246-3 (PMC12069139; doi:10.1186/s41118-025-00246-3)
Supplement: Supplementary file 1 — Supplementary material 1. [file 41118_2025_246_MOESM1_ESM.docx]

*Table A1*. Regression parameters for the 4-class model for men

|  | Term |  | Coef | S.E. | z | p |
| --- | --- | --- | --- | --- | --- | --- |
| Expectation(no) | Intercept | class(1) | -2.4363 | 0.9222 | -2.6418 | 0.0083 |
| Expectation(yes) | Intercept | class(1) | 2.3251 | 0.5111 | 4.5496 | 5.40E-06 |
| Expectation(unsure) | Intercept | class(1) | 0.1113 | 0.6158 | 0.1807 | 0.86 |
| Expectation(no) | B-spline(1) | class(1) | -0.6452 | 2.6897 | -0.2399 | 0.81 |
| Expectation(yes) | B-spline(1) | class(1) | 2.1431 | 1.5891 | 1.3486 | 0.18 |
| Expectation(unsure) | B-spline(1) | class(1) | -1.4979 | 1.6267 | -0.9208 | 0.36 |
| Expectation(no) | B-spline(2) | class(1) | -2.7788 | 1.656 | -1.678 | 0.093 |
| Expectation(yes) | B-spline(2) | class(1) | 0.3675 | 1.5159 | 0.2425 | 0.81 |
| Expectation(unsure) | B-spline(2) | class(1) | 2.4113 | 1.1374 | 2.12 | 0.034 |
| Expectation(no) | B-spline(3) | class(1) | 7.6852 | 1.7817 | 4.3135 | 1.60E-05 |
| Expectation(yes) | B-spline(3) | class(1) | -6.7704 | 2.0995 | -3.2247 | 0.0013 |
| Expectation(unsure) | B-spline(3) | class(1) | -0.9148 | 1.3498 | -0.6777 | 0.5 |
| Expectation(no) | B-spline(4) | class(1) | 10.9181 | 8.3978 | 1.3001 | 0.19 |
| Expectation(yes) | B-spline(4) | class(1) | -15.3239 | 16.4884 | -0.9294 | 0.35 |
| Expectation(unsure) | B-spline(4) | class(1) | 4.4059 | 8.2522 | 0.5339 | 0.59 |
| Parenthood | Intercept | class(1) | -25.0089 | 10.7233 | -2.3322 | 0.02 |
| Parenthood | B-spline(1) | class(1) | 25.6946 | 13.6322 | 1.8848 | 0.059 |
| Parenthood | B-spline(2) | class(1) | 19.5638 | 7.8884 | 2.4801 | 0.013 |
| Parenthood | B-spline(3) | class(1) | 27.8049 | 14.0522 | 1.9787 | 0.048 |
| Parenthood | B-spline(4) | class(1) | -28.2848 | 25.1836 | -1.1231 | 0.26 |
| Expectation(no) | Intercept | class(2) | -8.5664 | 5.4239 | -1.5794 | 0.11 |
| Expectation(yes) | Intercept | class(2) | 4.7864 | 2.7297 | 1.7535 | 0.08 |
| Expectation(unsure) | Intercept | class(2) | 3.78 | 2.7326 | 1.3833 | 0.17 |
| Expectation(no) | B-spline(1) | class(2) | 20.0459 | 14.9437 | 1.3414 | 0.18 |
| Expectation(yes) | B-spline(1) | class(2) | -10.8958 | 7.5061 | -1.4516 | 0.15 |
| Expectation(unsure) | B-spline(1) | class(2) | -9.1502 | 7.5214 | -1.2165 | 0.22 |
| Expectation(no) | B-spline(2) | class(2) | -41.2162 | 28.5982 | -1.4412 | 0.15 |
| Expectation(yes) | B-spline(2) | class(2) | 23.0893 | 14.3053 | 1.614 | 0.11 |
| Expectation(unsure) | B-spline(2) | class(2) | 18.1269 | 14.3535 | 1.2629 | 0.21 |
| Expectation(no) | B-spline(3) | class(2) | 49.7423 | 33.1356 | 1.5012 | 0.13 |
| Expectation(yes) | B-spline(3) | class(2) | -24.5369 | 16.5763 | -1.4802 | 0.14 |
| Expectation(unsure) | B-spline(3) | class(2) | -25.2054 | 16.6244 | -1.5162 | 0.13 |
| Expectation(no) | B-spline(4) | class(2) | -114.233 | 84.2297 | -1.3562 | 0.18 |
| Expectation(yes) | B-spline(4) | class(2) | 53.5517 | 42.1098 | 1.2717 | 0.2 |
| Expectation(unsure) | B-spline(4) | class(2) | 60.6814 | 42.1548 | 1.4395 | 0.15 |
| Parenthood | Intercept | class(2) | -6.5228 | 2.9407 | -2.2181 | 0.027 |
| Parenthood | B-spline(1) | class(2) | 0.5226 | 4.7082 | 0.111 | 0.91 |
| Parenthood | B-spline(2) | class(2) | 4.7606 | 3.093 | 1.5391 | 0.12 |
| Parenthood | B-spline(3) | class(2) | 5.774 | 3.7399 | 1.5439 | 0.12 |
| Parenthood | B-spline(4) | class(2) | 1.4822 | 3.4351 | 0.4315 | 0.67 |
| Expectation(no) | Intercept | class(3) | -2.7265 | 1.0899 | -2.5015 | 0.012 |
| Expectation(yes) | Intercept | class(3) | 0.7206 | 0.6234 | 1.1558 | 0.25 |
| Expectation(unsure) | Intercept | class(3) | 2.0059 | 0.558 | 3.5947 | 0.00033 |
| Expectation(no) | B-spline(1) | class(3) | 0.0725 | 1.7739 | 0.0409 | 0.97 |
| Expectation(yes) | B-spline(1) | class(3) | -0.0099 | 1.1394 | -0.0087 | 0.99 |
| Expectation(unsure) | B-spline(1) | class(3) | -0.0626 | 0.9432 | -0.0664 | 0.95 |
| Expectation(no) | B-spline(2) | class(3) | 4.9973 | 1.2849 | 3.8894 | 0.0001 |
| Expectation(yes) | B-spline(2) | class(3) | -3.1417 | 1.2515 | -2.5105 | 0.012 |
| Expectation(unsure) | B-spline(2) | class(3) | -1.8556 | 0.7993 | -2.3215 | 0.02 |
| Expectation(no) | B-spline(3) | class(3) | -1.1732 | 1.8336 | -0.6398 | 0.52 |
| Expectation(yes) | B-spline(3) | class(3) | 0.5259 | 1.8788 | 0.2799 | 0.78 |
| Expectation(unsure) | B-spline(3) | class(3) | 0.6474 | 1.1377 | 0.569 | 0.57 |
| Expectation(no) | B-spline(4) | class(3) | 8.3833 | 3.0934 | 2.7101 | 0.0068 |
| Expectation(yes) | B-spline(4) | class(3) | -9.5441 | 5.8149 | -1.6413 | 0.1 |
| Expectation(unsure) | B-spline(4) | class(3) | 1.1608 | 2.9665 | 0.3913 | 0.7 |
| Parenthood | Intercept | class(3) | -45.4697 | 35.6882 | -1.2741 | 0.2 |
| Parenthood | B-spline(1) | class(3) | 51.7026 | 43.8856 | 1.1781 | 0.24 |
| Parenthood | B-spline(2) | class(3) | 30.2824 | 29.2012 | 1.037 | 0.3 |
| Parenthood | B-spline(3) | class(3) | 47.4375 | 38.3205 | 1.2379 | 0.22 |
| Parenthood | B-spline(4) | class(3) | 38.8862 | 34.5627 | 1.1251 | 0.26 |
| Expectation(no) | Intercept | class(4) | 1.2061 | 0.6084 | 1.9823 | 0.047 |
| Expectation(yes) | Intercept | class(4) | -2.072 | 1.1283 | -1.8364 | 0.066 |
| Expectation(unsure) | Intercept | class(4) | 0.8659 | 0.6364 | 1.3606 | 0.17 |
| Expectation(no) | B-spline(1) | class(4) | -1.8635 | 1.7457 | -1.0675 | 0.29 |
| Expectation(yes) | B-spline(1) | class(4) | 2.8606 | 3.258 | 0.878 | 0.38 |
| Expectation(unsure) | B-spline(1) | class(4) | -0.9971 | 1.8819 | -0.5299 | 0.6 |
| Expectation(no) | B-spline(2) | class(4) | 6.0165 | 3.5364 | 1.7013 | 0.089 |
| Expectation(yes) | B-spline(2) | class(4) | -6.1752 | 6.5896 | -0.9371 | 0.35 |
| Expectation(unsure) | B-spline(2) | class(4) | 0.1587 | 3.823 | 0.0415 | 0.97 |
| Expectation(no) | B-spline(3) | class(4) | 3.3096 | 4.7422 | 0.6979 | 0.49 |
| Expectation(yes) | B-spline(3) | class(4) | -5.892 | 9.1787 | -0.6419 | 0.52 |
| Expectation(unsure) | B-spline(3) | class(4) | 2.5824 | 5.0918 | 0.5072 | 0.61 |
| Expectation(no) | B-spline(4) | class(4) | 0.7812 | 2.2709 | 0.344 | 0.73 |
| Expectation(yes) | B-spline(4) | class(4) | 2.4683 | 2.4919 | 0.9905 | 0.32 |
| Expectation(unsure) | B-spline(4) | class(4) | -3.2495 | 4.27 | -0.761 | 0.45 |
| Parenthood | Intercept | class(4) | -4.5467 | 1.8254 | -2.4908 | 0.013 |
| Parenthood | B-spline(1) | class(4) | 9.1153 | 8.1011 | 1.1252 | 0.26 |
| Parenthood | B-spline(2) | class(4) | -55.7668 | 38.7204 | -1.4402 | 0.15 |
| Parenthood | B-spline(3) | class(4) | 75.7232 | 50.9949 | 1.4849 | 0.14 |
| Parenthood | B-spline(4) | class(4) | -654.703 | 466.5913 | -1.4032 | 0.16 |

*Table A2*. Regression parameters for the 3-class model for women

|  | Term |  | Coef | S.E. | z | p |
| --- | --- | --- | --- | --- | --- | --- |
| Expectation(no) | Intercept | class(1) | -2.6796 | 0.7861 | -3.4088 | 0.00065 |
| Expectation(yes) | Intercept | class(1) | 2.7882 | 0.4322 | 6.4515 | 1.10E-10 |
| Expectation(unsure) | Intercept | class(1) | -0.1086 | 0.4578 | -0.2372 | 0.81 |
| Expectation(no) | B-spline(1) | class(1) | 1.696 | 1.8016 | 0.9414 | 0.35 |
| Expectation(yes) | B-spline(1) | class(1) | -0.4256 | 0.9826 | -0.4331 | 0.66 |
| Expectation(unsure) | B-spline(1) | class(1) | -1.2704 | 1.0194 | -1.2462 | 0.21 |
| Expectation(no) | B-spline(2) | class(1) | -5.502 | 2.4365 | -2.2581 | 0.024 |
| Expectation(yes) | B-spline(2) | class(1) | 2.546 | 1.304 | 1.9524 | 0.051 |
| Expectation(unsure) | B-spline(2) | class(1) | 2.956 | 1.2632 | 2.3401 | 0.019 |
| Expectation(no) | B-spline(3) | class(1) | 2.7638 | 1.2803 | 2.1586 | 0.031 |
| Expectation(yes) | B-spline(3) | class(1) | -2.5358 | 0.8386 | -3.0238 | 0.0025 |
| Expectation(unsure) | B-spline(3) | class(1) | -0.228 | 0.743 | -0.3069 | 0.76 |
| Expectation(no) | B-spline(4) | class(1) | 4.7873 | 0.891 | 5.3731 | 7.70E-08 |
| Expectation(yes) | B-spline(4) | class(1) | -5.612 | 0.8427 | -6.6596 | 2.70E-11 |
| Expectation(unsure) | B-spline(4) | class(1) | 0.8248 | 0.6147 | 1.3418 | 0.18 |
| Parenthood | Intercept | class(1) | -18.3553 | 5.9313 | -3.0946 | 0.002 |
| Parenthood | B-spline(1) | class(1) | 15.4181 | 7.0288 | 2.1936 | 0.028 |
| Parenthood | B-spline(2) | class(1) | 17.0413 | 5.1911 | 3.2828 | 0.001 |
| Parenthood | B-spline(3) | class(1) | 16.7137 | 6.4002 | 2.6114 | 0.0091 |
| Parenthood | B-spline(4) | class(1) | 15.131 | 5.7995 | 2.609 | 0.0091 |
| Expectation(no) | Intercept | class(2) | -34.0533 | 62.5844 | -0.5441 | 0.59 |
| Expectation(yes) | Intercept | class(2) | 17.1578 | 31.294 | 0.5483 | 0.58 |
| Expectation(unsure) | Intercept | class(2) | 16.8955 | 31.2914 | 0.5399 | 0.59 |
| Expectation(no) | B-spline(1) | class(2) | 30.9697 | 66.0973 | 0.4685 | 0.64 |
| Expectation(yes) | B-spline(1) | class(2) | -15.4988 | 33.05 | -0.4689 | 0.64 |
| Expectation(unsure) | B-spline(1) | class(2) | -15.4709 | 33.0519 | -0.4681 | 0.64 |
| Expectation(no) | B-spline(2) | class(2) | 32.7628 | 60.9664 | 0.5374 | 0.59 |
| Expectation(yes) | B-spline(2) | class(2) | -18.3318 | 30.5047 | -0.601 | 0.55 |
| Expectation(unsure) | B-spline(2) | class(2) | -14.431 | 30.478 | -0.4735 | 0.64 |
| Expectation(no) | B-spline(3) | class(2) | 33.7962 | 63.1912 | 0.5348 | 0.59 |
| Expectation(yes) | B-spline(3) | class(2) | -18.4952 | 31.6299 | -0.5847 | 0.56 |
| Expectation(unsure) | B-spline(3) | class(2) | -15.301 | 31.5926 | -0.4843 | 0.63 |
| Expectation(no) | B-spline(4) | class(2) | 39.8367 | 62.2621 | 0.6398 | 0.52 |
| Expectation(yes) | B-spline(4) | class(2) | -23.4056 | 31.3755 | -0.746 | 0.46 |
| Expectation(unsure) | B-spline(4) | class(2) | -16.4311 | 31.2215 | -0.5263 | 0.6 |
| Parenthood | Intercept | class(2) | -3.9056 | 0.7786 | -5.0161 | 5.30E-07 |
| Parenthood | B-spline(1) | class(2) | -0.3964 | 2.265 | -0.175 | 0.86 |
| Parenthood | B-spline(2) | class(2) | -3.9924 | 3.9254 | -1.0171 | 0.31 |
| Parenthood | B-spline(3) | class(2) | 5.3939 | 3.8912 | 1.3862 | 0.17 |
| Parenthood | B-spline(4) | class(2) | -11.0621 | 9.1627 | -1.2073 | 0.23 |
| Expectation(no) | Intercept | class(3) | 0.0227 | 0.2612 | 0.0869 | 0.93 |
| Expectation(yes) | Intercept | class(3) | -0.639 | 0.382 | -1.6726 | 0.094 |
| Expectation(unsure) | Intercept | class(3) | 0.6162 | 0.2579 | 2.3891 | 0.017 |
| Expectation(no) | B-spline(1) | class(3) | -0.023 | 0.6842 | -0.0336 | 0.97 |
| Expectation(yes) | B-spline(1) | class(3) | -0.5815 | 1.1057 | -0.5259 | 0.6 |
| Expectation(unsure) | B-spline(1) | class(3) | 0.6045 | 0.7863 | 0.7688 | 0.44 |
| Expectation(no) | B-spline(2) | class(3) | 0.6745 | 1.0711 | 0.6297 | 0.53 |
| Expectation(yes) | B-spline(2) | class(3) | 1.4593 | 1.7288 | 0.8441 | 0.4 |
| Expectation(unsure) | B-spline(2) | class(3) | -2.1338 | 1.3062 | -1.6336 | 0.1 |
| Expectation(no) | B-spline(3) | class(3) | 3.4703 | 1.8863 | 1.8397 | 0.066 |
| Expectation(yes) | B-spline(3) | class(3) | -5.3912 | 3.188 | -1.6911 | 0.091 |
| Expectation(unsure) | B-spline(3) | class(3) | 1.9209 | 2.1658 | 0.8869 | 0.38 |
| Expectation(no) | B-spline(4) | class(3) | 11.0441 | 5.7359 | 1.9254 | 0.054 |
| Expectation(yes) | B-spline(4) | class(3) | 3.9494 | 7.6132 | 0.5188 | 0.6 |
| Expectation(unsure) | B-spline(4) | class(3) | -14.9934 | 10.0685 | -1.4891 | 0.14 |
| Parenthood | Intercept | class(3) | -121.194 | 153.2813 | -0.7907 | 0.43 |
| Parenthood | B-spline(1) | class(3) | 126.9566 | 162.4742 | 0.7814 | 0.43 |
| Parenthood | B-spline(2) | class(3) | 110.3946 | 148.3322 | 0.7442 | 0.46 |
| Parenthood | B-spline(3) | class(3) | 118.6469 | 154.6297 | 0.7673 | 0.44 |
| Parenthood | B-spline(4) | class(3) | 117.9395 | 152.9672 | 0.771 | 0.44 |
